# Reference Values of Skin Autofluorescence by Age Groups in Healthy Spanish Adults: Results from the EVasCu Study, a Systematic Review, and a Meta-Analysis

**DOI:** 10.3390/jcm14020474

**Published:** 2025-01-13

**Authors:** Irene Martínez-García, Iván Cavero-Redondo, Carlos Pascual-Morena, Iris Otero-Luis, Marta Fenoll-Morante, Carla Geovanna Lever-Megina, Eva Rodríguez-Gutiérrez, Alicia Saz-Lara

**Affiliations:** 1 CarVasCare Research Group (2023-GRIN-34459), Faculty of Nursing, Universidad de Castilla-La Mancha, 16002 Cuenca, Spain; irene.mgarcia@uclm.es (I.M.-G.); ivan.cavero@uclm.es (I.C.-R.); iris.otero@uclm.es (I.O.-L.); marta.fenoll@alu.uclm.es (M.F.-M.); carlageovanna.lever@alu.uclm.es (C.G.L.-M.); alicia.delsaz@uclm.es (A.S.-L.); 2 Health and Social Research Center, Universidad de Castilla-La Mancha, 16002 Cuenca, Spain; eva.rodriguez@uclm.es; 3 Facultad de Enfermería de Albacete, Universidad de Castilla-La Mancha, 02008 Albacete, Spain; 4 Research Network on Chronicity, Primary Care and Health Promotion (RICAPPS), 16071 Cuenca, Spain

**Keywords:** advanced glycation end products, skin autofluorescence, healthy adults, reference values, age, sex, smoking status, skin phototype

## Abstract

**Background/Objectives**: Age is a known predictor of skin autofluorescence (SAF) across populations, but age-based reference values are lacking for the Spanish population. This study aims to establish SAF reference values for healthy Spanish adults by age group, compare these with other populations, and estimate optimal SAF cut-off points by age range. Additionally, it aims to analyse the influence of sex, smoking, and skin phototype. **Methods**: This cross-sectional EVasCu study included 390 healthy subjects aged over 18 years. Participants’ age, sex, smoking status, and skin were recorded and categorised into age groups. Advanced glycation end products were measured through the SAF. A systematic review and meta-analysis, including an EVasCu study, was performed to obtain pooled means and standard deviations by age group. **Results**: The mean SAF Spanish values by age were (95% CI): (i) 18–19 years: 1.34–1.56 arbitrary units (AU); (ii) 20–29 years: 1.56–1.70 AU; (iii) 30–39 years: 1.66–1.84 AU; (iv) 40–49 years: 1.79–1.91 AU; (v) 50–59 years: 2.07–2.21 AU; (vi) ≥60 years: 2.07–2.50 AU. SAF was significantly correlated with age (r = 0.531; *p* < 0.001), smoking status (r = −0.196; *p* < 0.001), and skin phototype (r = 0.138; *p* = 0.007), and SAF was greater in smokers and dark-skinned individuals (*p* < 0.05). No significant differences were found in the SAF values for sex. The results of the meta-analysis were in line with those of the present study, providing reference values of SAF for the general population. **Conclusions**: SAF increases linearly with age in healthy individuals, and higher levels of SAF are observed in smokers and dark-skinned individuals.

## 1. Introduction

Advanced glycation end products (AGEs) were first defined by Maillard in 1912, who described their formation by non-enzymatic chemical reactions between the carbonyl groups of reducing sugars and amino acids [1]. It was later discovered that these products could be produced and accumulated via other pathways, such as the autoxidation of sugars (glyco-oxidation) or the peroxidation of fatty acids (lipo-oxidation), generating reactive intermediates and non-enzymatic processes that cause the consequent modification of proteins, lipids, and nucleic acids [2,3,4,5]. These glycation and oxidation processes are natural chemical reactions that occur constantly in our bodies as part of metabolism; however, they can increase under conditions of oxidative stress and hyperglycaemia, increasing the formation and accumulation of AGEs [6]. The excessive accumulation of advanced glycation end products (AGEs) favours further interactions with their specific receptors (RAGE), activating inflammatory and prooxidative pathways that contribute to the development and progression of various chronic diseases [2,3].

Due to their ability to emit fluorescence, it is possible to distinguish between fluorescent AGEs, such as pentosidine, and non-fluorescent AGEs, such as carboxymethyllysine (CML) [2]. In early studies, AGEs were measured by their collagen-bound fluorescence (CLF) or specific measurements of AGEs (pentosidine, carboxymethyllysine) via laboratory tests on skin biopsies [7,8,9]. These parameters are subsequently determined via high-performance liquid chromatography, enzyme-linked immunosorbent assay, or serum fluorescence in plasma [10,11]. However, plasma AGE assays are difficult to reproduce and correlate poorly with tissue AGE levels. Owing to the autofluorescence of most of these products, it is now possible to determine AGEs via skin autofluorescence (SAF), a non-invasive method that provides an immediate estimate of AGE accumulation and has been previously validated [12,13,14]. Skin autofluorescence (SAF) is a technique that measures the fluorescence emitted by advanced glycation end products (AGEs) when exposed to specific wavelengths of light [14]. The use of SAF (typically on the forearm) as a surrogate for the assessment of total systemic AGE levels makes it a non-invasive, rapid, and simple method of assessment [13,14].

Maillard was the first to link ageing to the accumulation of AGEs [1], a hypothesis subsequently confirmed by numerous studies, which also correlated them with increased cardiovascular risk due to their detrimental effects on the vascular wall, especially in the population with diabetes [15,16,17]. Currently, the prevalence of cardiovascular disease (CVD) is increasing worldwide [18], and CVD is the leading cause of death in the Spanish population, according to the National Statistics Institute [19]. The increase in the incidence of CVD is associated with several key risk factors. These include the progressive ageing of the population [20], a phenomenon that is particularly evident in developed countries. This is due to declining birth rates, increased life expectancy, improved sanitary conditions, and quality of healthcare, including vaccination and treatment of acute infections [20,21,22]. In addition to these factors, male sex and smoking are recognised as risk factors for CVD [23].

The characteristics of the SAF tool, a non-invasive, rapid, and simple technique for early detection, could represent a breakthrough if it was introduced into clinical practice as a screening method to meet the needs of the healthcare system for CVD and associated complications [24,25,26]. However, it can be difficult for healthcare professionals to interpret the SAF due to the variability of the test, as several factors influence the results [27,28,29]. In diabetes mellitus, AGE formation is accelerated by the hyperglycemia and oxidative stress characteristic of this disease [15], which are closely correlated with age and the severity of diabetic complications [9]. In a healthy population, with no diagnosed chronic diseases and without medication, age is the main determinant of AGEs accumulation, but other variables, such as smoking and sex, can significantly affect AGEs accumulation [29,30]. When measuring AGEs via the SAF, the skin phototype is a factor to consider, as it may influence the variability of results [31]. In addition, the type of population or ethnicity may influence the variability of the SAF score, even after adjusting for potential confounding variables [32]. Establishing reference values for the SAF score in healthy subjects is essential to facilitate the interpretation of results in different pathological settings and to identify possible pathological findings that may be associated with risks or pathologies related to advanced glycation. Previous studies have established reference values in healthy subjects from different populations, such as Dutch and Chinese populations [29,33]. However, there are currently no published reference values for SAF in healthy individuals (i.e., without diagnosed chronic diseases and without medication) residing in Spain, with age being considered the main confounding variable, which hinders the diagnostic accuracy of SAF and the correct interpretation of results by healthcare professionals, making it difficult. A meta-analysis of SAF in different age ranges could be key to establishing accurate and personalised clinical criteria in healthy populations, thus improving prevention, diagnosis, and lifelong CVD.

Therefore, the main objectives of this study were as follows: (1) to establish reference values for the SAF score in healthy Spanish subjects over 18 years of age, categorised by age group; (2) to compare the results with reference values in other previously studied populations; and (3) to estimate optimal cut-off points for the SAF score in different age ranges in a healthy general population. In addition, a secondary objective was to analyse the influence of other factors, such as sex, smoking status, and skin phototype, on the variation in SAF values in healthy subjects.

## 2. Materials and Methods

### 2.1. EVasCu Study

#### 2.1.1. Study Design and Participants

This study is based on data from the cross-sectional EVasCu study in Spain, which was designed to validate a model of early vascular ageing (EVA) as an index of cardiovascular risk in healthy adults using confirmatory factor analysis [34]. This study was approved by the Clinical Research Ethics Committee of the Cuenca Health Area (REG: 2022/PI2022) and was conducted according to the guidelines for reporting observational studies, “Strengthening the Reporting of Observational Studies in Epidemiology (STROBE) Statement” (Supplementary Table S1) [35], and the standards of the Declaration of Helsinki.

#### 2.1.2. Study Sample

The sample size was calculated using Epidat software (version 4.2), which yielded a sample size of 355 healthy participants, an estimated effect of 1, an alpha risk of 0.05, and an absolute precision level of 0.04 to detect a statistically significant result for the EVA index [36]. Finally, 390 participants were enrolled.

The participants volunteered for the study after the project was advertised through various media and social networks. The inclusion criteria were as follows: over 18 years of age, clinically stable for the previous 6 weeks, and provided written consent to participate in the study. Subjects enrolled in other trials of any intervention, those receiving pharmacological treatment, or those with a previous diagnosis of CVD (such as diabetes, arterial hypertension, myocardial infarction, or stroke) were excluded from the study. The recruitment and measurement period lasted from June 2022 to December 2022 at the Health and Social Research Centre of the University of Castilla-La Mancha.

#### 2.1.3. Dependent Variable

AGEs were considered the dependent variable and were measured along the SAF using the AGE Reader (DiagnOptics Technologies BV, Groningen, The Netherlands). This device performs three consecutive individual measurements by autofluorescence, providing a more accurate and reliable final average of the measurements. Measurements were taken on both arms, avoiding skin imperfections, such as scars, moles, tattoos, and birthmarks, and the resulting average was taken from both arms. The participants were asked not to use any skin cream before the measurement to avoid interference with the results [37]. In addition, the light in the room was controlled, and a dark room was chosen for the measurements to avoid interference from any external light source. The SAF values are expressed in arbitrary units (AU).

#### 2.1.4. Independent Variables

Age data were obtained from self-reported questionnaires. Age was divided into six categories according to the literature reviewed [29,38] to ensure consistency and to facilitate comparability of results: (i) 18–19 years; (ii) 20–29 years; (iii) 30–39 years; (iv) 40–49 years; (v) 50–59 years; and (vi) 60 years and older.

#### 2.1.5. Other Variables

Sex and smoking status were collected through self-reported questionnaires. Sex was classified as women or men, and smoking status was classified as follows: (i) smoker; (ii) ex-smoker; and (iii) non-smoker.

Skin phototype was assessed using the Fitzpatrick Skin Type (FST), which classifies the skin phototype into six categories: (i) FST I: light/pale white; (ii) FST II: white/fair; (iii) FST III: medium/white to olive; (iv) FST IV: olive/moderate brown; (v) FST V: brown/dark brown; and (vi) FST VI: black/very dark brown to black [39]. The skin phototypes were then divided into three categories to increase the intragroup size, reduce complexity, and simplify the interpretation of the results due to the similarity of their characteristics: (i) FST I-II; (ii) FST III-IV; and (iii) FST V-VI.

#### 2.1.6. Statistical Analysis

Normal probability plots and the Kolmogorov–Smirnov test were used to test the normality of the distribution of continuous variables. Descriptive data for the total sample are presented as the means and standard deviations (SDs) or numbers of subjects and proportions (%), as appropriate.

Age and skin phototype were categorised as previously described. Pearson correlation coefficients were calculated for the SAF score and each of the independent variables. Linear regression was performed between the SAF score and age to obtain the slope of the regression and the coefficient of determination (R^2^). The general linear model was used to study the effect of age on SAF levels, generating an equation for SAF and a new variable on the basis of this equation. To analyse the predictive and validating capacity of the equation, the association between the actual dependent variable and the one estimated by the equation was evaluated. Student’s *t*-tests or two-way ANOVAs were then performed, depending on the number of categories in the independent variables (age, sex, smoking status, and FST), to determine the existence of statistically significant differences between the groups. The missing data were not considered in the analyses. A *p*-value < 0.05 was considered statistically significant. SPSS version 16 for Windows (SPSS Inc., Chicago, IL, USA) was used for the statistical analyses.

### 2.2. Systematic Review and Meta-Analysis

The systematic review and meta-analysis were conducted in accordance with the PRISMA statement for systematic reviews and meta-analyses (Supplementary Table S2) [40], the MOOSE (Meta-analysis of Observational Studies in Epidemiology) statement (Supplementary Table S3) [41], and the recommendations of the Cochrane Collaboration Handbook [42]. This study was registered in the International Prospective Register of Systematic Reviews (PROSPERO: CRD42024519272).

#### 2.2.1. Search Strategy

The systematic search was conducted in three databases, Scopus, PubMed, and Web of Science, from inception to April 2024. The search strategy is described in Supplementary Table S4. In addition, we searched the grey literature (Google Scholar, Theseo, Networked Digital Library of Theses and Dissertations, and OpenGrey), reference organisations, and websites, as well as manual searches of the bibliographic references of the selected articles, systematic reviews, or previous meta-analyses that were performed. The references identified were included in the reference manager used (Mendeley, version 1.19.6).

The systematic search was performed independently by two reviewers (IMG and CPM), and disagreements were resolved by consensus or by a third reviewer (ICR).

#### 2.2.2. Inclusion/Exclusion Criteria

The inclusion criteria were as follows: (i) participants: healthy population over 18 years of age without previously diagnosed pathologies; (ii) design: cross-sectional studies, including cross-sectional studies derived from other types of studies; (iii) exposure: SAF had to be available according to the age of the participants; and (iv) outcome: SAF.

The exclusion criteria were as follows: (i) participants: studies that included underage participants and could not be excluded from the analysis; (ii) studies that did not classify SAF by established age groups or studies without the ability to extract these data; and (iii) studies that were not written in English or Spanish.

Study selection was performed independently by two reviewers (IMG and CPM), and disagreements were resolved by consensus or by a third reviewer (ICR).

#### 2.2.3. Data Extraction

An ad hoc table was created with the following data from the included studies: (i) reference; (ii) country; (iii) study design; (iv) sample size; (v) mean age; (vi) age ranges; (vii) percentage of smokers; (viii) percentage of females; (ix) skin phototypes assessed by the FST; and (x) outcome (SAF).

#### 2.2.4. Quality Assessment

The quality of the studies was assessed using the Quality Assessment Tool for Observational Cohort and Cross-Sectional Studies from the US National Institutes of Health National Heart, Lung, and Blood Institute [43]. This tool assesses 14 items, each of which is rated individually as “yes”, “no”, “cannot determine”, “not reported” or “not applicable”. To assess the overall quality, each item is evaluated individually, without a specific formula for calculating a total score based on the number of items rated as ‘poor’. Instead, the study is assessed holistically, taking into account its strengths and weaknesses across all 14 items, which are rated as good, fair, or poor.

The quality of the studies was assessed independently by two reviewers (IMG and CPM), and disagreements were resolved by consensus or by a third reviewer (ICR).

#### 2.2.5. Statistical Analysis

A narrative synthesis of the systematic review was performed. Exceptionally, WebPlotDigitizer 4.7 software was used to extract data from the graphs in the primary studies. When the primary studies did not include a ‘60 years and older’ age group but reported data by groups of 10-year age ranges, the pooled mean and pooled standard deviation were calculated from the primary data using the data pooling formulas (Supplementary Figure S1).

Random-effects meta-analysis was performed by the DerSimonian and Laird method, and the mean and standard error were used to estimate the weight of each study for each age group (95% CI) [44]. A subgroup analysis was performed according to the smoking status of the participants in each established age group, considering ex-smokers within the group of non-smokers. Heterogeneity was measured by I^2^ and was classified as not important if it was <30%, moderate if it was 30–50%, substantial if it was 50–75%, and considerable if it was >75% [45,46]. Sensitivity analysis (systematic reanalysis by eliminating studies one at a time) was performed to assess the robustness of the summary estimates. Publication bias was assessed using the Egger test, with a *p*-value < 0.1 suggesting the presence of publication bias [47].

Stata SE software, version 15 (StataCorp, College Station, TX, USA), was used for statistical analyses.

## 3. Results

### 3.1. EVasCu Results

Of the 390 subjects, 386 had SAF values because the AGE Reader Mu^®^ was unable to measure values in four subjects with low cutaneous skin reflexes (dark skin phototype) (Supplementary Figure S2). The mean age was 42.13 ± 13.14 years (62.95% females).

The characteristics of the study participants by age groups are presented in Table 1, where the dependent variable is SAF and the independent variables are sex, smoking status, and FST. The mean reference values of SAF in healthy Spanish subjects for each age group were as follows: (i) 18–19 years: 1.34–1.56 arbitrary units; (ii) 20–29 years: 1.56–1.70 AU; (iii) 30–39 years: 1.66–1.84 AU; (iv) 40–49 years: 1.79–1.91 AU; (v) 50–59 years: 2.07–2.21 AU; and (vi) 60 years and older: 2.07–2.50 AU.

A statistically significant correlation was found between age and SAF (r = 0.560; *p* < 0.001), and after age was categorised into groups, this significant correlation remained with SAF (r = 0.528; *p* < 0.001), showing an upward linear trend between SAF and age (Supplementary Figures S3 and S4). Furthermore, ANOVA revealed statistically significant differences between the SAF score and age groups (F = 32.38; *p* < 0.001).

Regardless of age, significant differences were found for smoking status and skin phototype but not for sex. After the influence of sex on SAF for each age group was analysed, no significant differences were found between men and women in the different groups, except for those 60 years and older (*p* < 0.05). For smoking status, significant differences were found only in the 20–29 years age group. For the skin phototype, significant differences were found in the 50–59 years age group (Supplementary Figures S5–S7).

After performing a linear regression analysis, the prediction equation for the SAF score as a function of age was generated for the healthy Spanish population over 18 years of age, which is presented below:

SAF = 1.149 + 0.018 × age (R^2^= 31.4%; F = 175.40, *p* < 0.001), (1)

The following equation was used to create a new variable to predict SAF from age. After correlation analysis, a statistically significant association was found between the actual SAF variable and the one created from the equation (r = 0.560 **, *p* < 0.001). The generated SAF variable obtained an overall mean of 1.90 ± 0.22 AU, indicating a slight overestimation compared with the actual SAF mean. Despite this discrepancy, the generated equation had a robust predictive ability, as a correlation was found between the actual and estimated variable, supporting the overall validity of the equation for use as an estimation tool for the SAF, specifically for the healthy Spanish population. In addition, sex, smoking status, and skin phototype were considered to assess how the equation might differ for each of these variables (Supplementary Table S5).

### 3.2. Systematic Review

#### 3.2.1. Characteristics of the Included Studies

**Figure 1:**
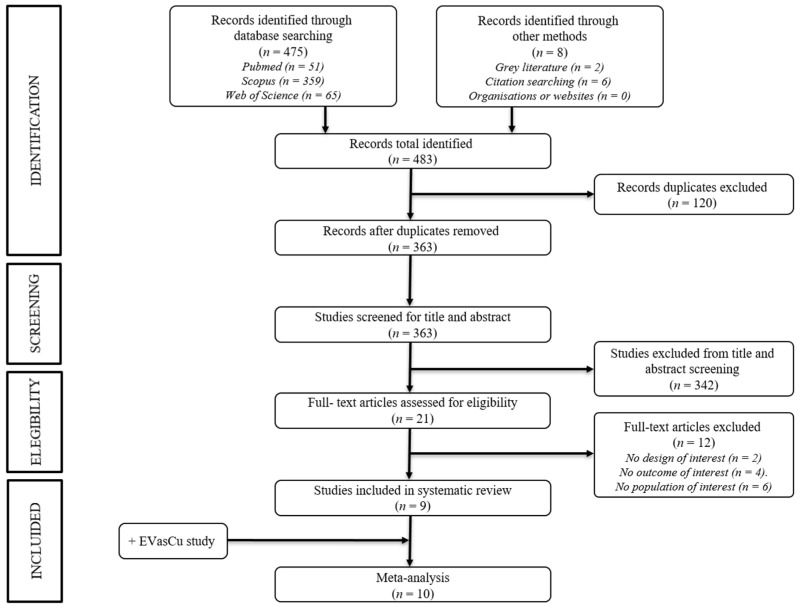
PRISMA flowchart of study selection.

The systematic search identified 475 records, of which the full texts of 21 were examined. After excluding 12 studies, nine studies were included in the systematic review [29,33,38,48,49,50,51,52,53] and meta-analysis, along with our study, the EVasCu study (Figure 1).

Of the ten included studies, three were from the Netherlands [29,38,52], two from Brazil [48,53], one from Germany [50], one from Slovakia [51], one from China [33], one from Japan [49], and one from Spain (EVasCu study) [35]. The studies were published between 2006 and 2024 and included a total of 15,288 subjects aged 0–96 years; all of them categorised SAF by age according to the established age groups (10-year intervals) or reported graphs between the two variables from which this information could be extracted. All the studies were conducted in the general population, including healthy participants; however, four studies also included a population with some pathology and reported these data separately from the healthy population. These studies included participants with chronic kidney disease, insulin resistance, diabetes, stroke, hypertension, dyslipidaemia, and medicated patients, and data from these participants were excluded from our analysis. Some studies reported the sex of the participants (by age group or/and in general), some reported the smoking status (by age group or/and in general), and others reported the skin phototype of the participants overall, assessed by the Fitzpatrick scale or skin reflectance (R%). The characteristics of the studies are shown in Table 2, which includes data from only the population considered healthy (with no diagnosed chronic diseases and without medication).

#### 3.2.2. Quality Assessment

According to the Quality Assessment Tool for Observational Cohort and Cross-Sectional Studies from the US National Institutes of Health National Heart, Lung, and Blood Institute, all studies were rated as low risk (or good quality). However, items 7, 10, and 13 were deemed not applicable, as they were cross-sectional studies without follow-up. With respect to item 12, none of the studies reported blinding. Additionally, for item 5, only 2 studies reported information on sample size calculations or descriptions of power. The risk of bias assessment is shown in Supplementary Table S6.

#### 3.2.3. Associations Between Skin Autofluorescence and Age Categorised into Groups

**Figure 2:**
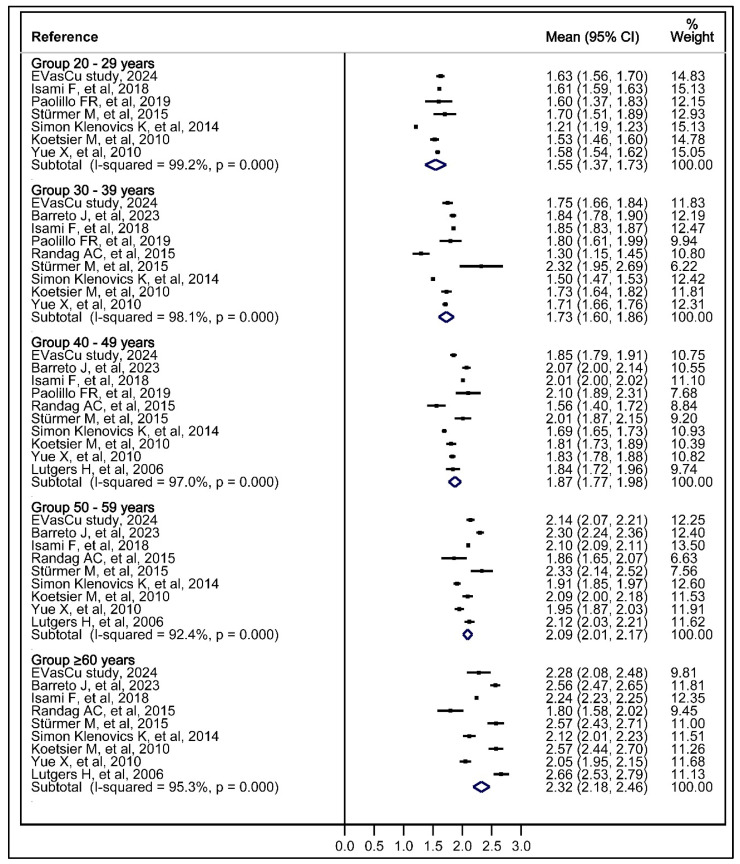
Meta-analysis of the mean skin autofluorescence by age groups [29,33,38,48,49,50,51,52,53].

Meta-analysis was performed by combining data for the 60 years and older of age group when the primary studies categorised this age group by 10-year ranges using the pooled mean and pooled standard deviation formula (Figure 2, Supplementary Figure S8). The meta-analysis provided estimates of the overall mean SAF for different age groups and their corresponding 95% confidence intervals (CIs): (i) 20–29 years: 1.55 (1.37, 1.73) AU; (ii) 30–39 years: 1.73 (1.60, 1.86) AU; (iii) 40–49 years: 1.87 (1.77, 1.98) AU; (v) 50–59 years: 2.09 (2.01, 2.17) AU; and (vi) 60 years and older: 2.32 (2.18, 2.46) AU. A trend toward increasing SAF with increasing age was observed, with significant differences found between the age groups of 40–49 years and 50–59 years, as well as between the age groups of 50–59 years and 60 years and older.

The figure shows a forest plot displaying the results of a meta-analysis based on the means and confidence intervals of SAF expressed in arbitrary units (AU) across age groups for each study. The % weight reflects the relative contribution of each study to the pooled effect estimate for each age group, influenced by the sample size and variability of the study. The I-squared statistic quantifies the heterogeneity between studies. Each point represents an individual study’s effect estimate, with bars indicating the 95% confidence interval (CI). The diamond shape shows the pooled effect estimate for each age group.

In addition to the EVasCu study, five studies with data stratified by smokers and non-smokers were included in the subgroup analysis [38,48,50,51,53]. Subgroup analyses showed a non-statistically significant positive trend between smoking and the mean SAF score for similar age groups in most studies, although it was statistically significant in one study, for participants aged 30–39 years and for participants aged 60 years and older [38]. The results of the meta-analysis were consistent with these findings, with an overall trend toward greater SAF in smokers than in non-smokers for similar age groups, but the difference did not reach statistical significance (Supplementary Table S7, Figure S9).

Heterogeneity was considerable for all age groups when not stratified by smoking status (I^2^ = 92.4–99.2%; *p* = 0.000), while when stratified, there were some exceptions with unimportant and substantial heterogeneity.

#### 3.2.4. Sensitivity Analysis

The pooled estimates of the means SAF categorised into age groups did not change significantly (in magnitude or direction) when individual study data were removed from the analysis one by one.

#### 3.2.5. Publication Bias

Finally, no evidence of publication bias was found using Egger’s test for the means of the SAFs categorised into age groups (*p*-value of the 20–29 years age group: 0.536; *p*-value of the 30–39 years age group: 0.592; *p*-value of the 40–49 years age group: 0.135; *p*-value of the 50–59 years age group: 0.872; *p*-value of the 60 years and older age group: 0.375).

## 4. Discussion

To our knowledge, this is the first study to provide baseline levels of SAF in healthy Spanish adults from the EVasCu study by age range, generating a linear prediction equation for SAF specific to this population considering age, which differs from those provided by previous studies for other non-Spanish populations [29,33,38,48,49,51]. Data from our cross-sectional study showed a significant correlation between SAF and age, smoking status, and FST, with higher levels of SAF found with increasing age, in smokers and/or ex-smokers, and in darker FST. However, no statistically significant association was found between SAF and sex.

With respect to age, the studies included in the systematic review are consistent with our data, with an increasing linear trend between SAF and age in healthy subjects, as shown in the meta-analysis by age groups [29,33,38,48,49,50,51,52,53]. A trend of increasing SAF with increasing age was observed, with significant differences between the 40–49 and 50–59 age groups and between the 50–59 years and 60 years and older age groups. These differences in older age groups may be due to the fact that the accumulation of AGEs in the skin increases with age, as the production of AGEs is a continuous process. In addition, oxidative stress increases with age, and the body’s ability to eliminate these products decreases and deteriorates [17,54,55]. An example of this is the decreased activity of the glyoxalase system, which reduces the detoxification of methylglyoxal, a precursor of AGEs in the skin, and therefore increases their formation [55,56,57]. Age-related collagen modifications, such as mineralisation and AGEs modification, reduce susceptibility to matrix metalloproteinases, preventing tissue remodelling and collagen degradation [54], which explains the increase in cutaneous AGEs with age. Furthermore, with age, a loss or dysfunction of physiological functions occurs, leading to a high prevalence of chronic diseases, which are particularly correlated with increased levels of AGEs [58,59]. Finally, external lifestyle factors such as smoking or diet may play a key role in the accumulation of AGEs [30,49], and the effects of an unhealthy lifestyle may accumulate as people age.

In terms of sex, no significant association was found between sex and SAF in the population studied in our study, which could be due to similarities in lifestyle and environmental factors between the sexes in the Spanish population. When the remaining included studies were considered, most did not report the SAF considering the sex of the participants for each age group. In addition, one of the included studies was conducted in women only [38] and reported a lower level of SAF than the other studies, including men. For these reasons, it was decided not to perform a subgroup analysis by sex. Although there is previous evidence that sex does not have a large effect on the variability of the SAF in European populations, there are differences in other populations, such as Arab or Eastern Mediterranean populations [32]; therefore, it would be advisable to report data disaggregated according to sex, ethnicity, lifestyle, and social determinants of the health of individuals in future studies.

Analysing the smoking status in our study, we found a significant association between smoking and SAF in the population studied, with the individuals with the highest levels of accumulation being smokers, followed by ex-smokers, and non-smokers being the individuals with the lowest SAF. The findings are important because smoking is a modifiable risk factor, underscoring the potential for intervention in the field of cardiovascular health. Our data are consistent with previous evidence that smoking directly contributes to increased SAF, since it is an exogenous source of AGEs and induces oxidative stress by increasing the amount of oxygen free radicals in the body [33]. For the remaining studies, smoking was reported differently in the collected studies, and most included ex-smokers in the non-smoker category. In addition, one study did not report smoking status [49], two studies included a non-smoking population without specifying whether ex-smokers were present [48,53], and only one study included never-smokers [38]. These factors could explain the high heterogeneity in the meta-analysis by smoking status subgroups, since it was performed by categorising smoking status into two groups, smokers and non-smokers, including ex-smokers in the non-smoker category. This explains the lack of significant differences, as a distinction should be made between ex-smokers and never-smokers, as recommended by Randag et al. [38]. Furthermore, as this is a self-reported variable that requires recall of consumption in terms of the number of cigarettes and years of smoking and is considered an unhealthy factor, there may be a reporting bias.

Regarding skin phototype, a significant association was found between skin phototype and SAF, with higher levels of accumulation observed in darker skin phototypes. After reviewing the studies included in the meta-analysis, it was not possible to perform a subgroup analysis because most studies reported the total skin phototypes included but did not report SAF values by age group, which would have allowed this covariate to be considered. Most studies have reported skin phototypes with FST, but it would be useful to assess and report the skin reflectances of subjects, as lower R% ranges (below 10–12%) could influence the variability of the SAF [31,33]. Recently, a new algorithm has been developed to calculate the SAF independently of skin colour [60], but it is currently unable to estimate the SAF in skins with very low skin reflectance, preventing measurements in Black subjects with very dark skin [31,61,62].

Currently, there are no cost-utility or cost-benefit studies addressing the potential introduction of the SAF as a screening test in the healthcare system. However, numerous studies have evaluated the efficacy of this test in detecting vascular complications, highlighting the high sensitivity of the SAF in detecting patients at risk and its possible use as a predictor of cardiovascular risk [28,58,59]. Considering the reference values for SAF in healthy subjects, high levels may indicate metabolic dysfunction, which may help the clinician to suspect possible undiagnosed disease and lead to further clinical evaluation. Due to this and the characteristics of the device, the SAF could be used as a screening test for cardiovascular risk, as it could provide fast and accurate results directly at the point of care, facilitating immediate clinical decision-making, improving care management, and reducing the waiting time for diagnosis. Our study helps to clarify the variability of the SAF test by providing age-dependent reference values for healthy Spanish subjects, as well as assessing other possible influencing factors, thus facilitating the clinical interpretation of the test results.

This study may have several clinical and research implications. First, it provides mean SAF values by age range in the Spanish population, as well as a general formula for estimating the SAF score in the healthy Spanish population based on age. Moreover, the results of the meta-analysis provide mean values and confidence intervals for healthy individuals, regardless of the type of population. It follows that abnormally elevated SAF values may indicate cardiovascular risk or undiagnosed CVD in apparently healthy individuals. The ability to detect this risk or CVD in its early stages would facilitate intervention on modifiable risk factors, thus facilitating the implementation of personalised prevention strategies (e.g., promotion of lifestyle changes) and timely medical treatment, which would prevent cardiovascular complications and improve quality of life. In addition, some variability in SAF estimates was explained, including skin phototype and smoking status. Our results are consistent with other studies, at least in terms of the trend observed; however, studies of SAF according to population ethnicity, which is also known to influence cardiovascular risk, are needed. In addition, it would be advisable for future studies to report in depth on smoking, analysing non-smokers and ex-smokers separately, since it is a modifiable factor that can significantly influence SAF.

However, we found several limitations that need to be considered. The data for the EVasCu study were derived from a cross-sectional study, and the other included studies were also cross-sectional in nature, which entails certain limitations, such as the inability to establish causal relationships and the potential for confounding effects due to the lack of temporal dimensions. Although the study was conducted in a ‘healthy population’ with no diagnosed medical conditions or prescriptions, there is a possibility that some individuals may have undiagnosed conditions such as diabetes or CVD, particularly in older adults who may manifest these conditions subclinically due to the cumulative risk associated with age. In addition, the possibility that the criteria for considering the population healthy may differ slightly between different studies is not ruled out. While the sample in the EVasCu study was limited in size, it was deemed sufficient for the initial analysis. However, to address the issue of sample size and enhance the robustness of the findings, a systematic review and subsequent meta-analysis were conducted, improving the overall statistical power of the analysis. Some studies identified during the systematic review could not be included because they did not report SAF data by age group but rather reported them overall or by tertile, which prevented comparisons between studies. In the meta-analysis, considerable heterogeneity was found between studies for all age groups due to differences in sample size and participant characteristics, as well as the reporting of covariates by authors, as described above. Moreover, the utilisation of disparate versions of the device and the employed software may give rise to heterogeneity in the outcomes of the meta-analysis, thereby introducing further variability that could impact the comparability and consistency of the analysed data. Furthermore, a variety of additional factors related to the individual (e.g., ethnicity, genetic determinants, family history of CVD, body mass index, physical activity, dietary variation, alcohol consumption, exposure to environmental pollutants, etc.) or to the test measurement (temperature, humidity, ambient light, etc.) have the potential to influence the variability of SAF [14,32,33,38,51,63] (Table S8). However, the aforementioned variables were not reported in the studies. It is recommended that these factors be analysed and reported according to age in future studies, and the importance of large-scale studies to unravel the contribution of each factor to the variability of the SAF is emphasised.

## 5. Conclusions

The results of our study showed that there is a linear association between SAF and age, with higher values found with increasing age in smokers and ex-smokers, and in healthy Spanish dark-skinned. Furthermore, the results of the meta-analysis were in line with those of our study, which included different populations. The mean SAF reference values established in this study could be used in Spanish populations in different clinical settings. In addition, an equation for the prediction of SAF in the healthy Spanish population is provided. Future research should focus on the evaluation of additional factors that may affect SAF.

## Figures and Tables

**Table 1:** Skin autofluorescence for each age group.

Variables	Age Groups (Years)						
	18–19	20–29	30–39	40–49	50–59	≥60	All Years
N	13	75	55	115	100	28	386
Men/Women	3/10	22/53	30/25	46/69	29/71	13/15	143/243
Smoke/Ex-smoker/Non-smoker	2/1/10	12/4/58	5/5/45	17/25/72	10/45/44	2/13/13	48/93/242
FST I-II/FST III-IV/FST V-VI	10/2/1	39/29/2	28/25/0	57/56/1	54/41/3	15/11/0	203/164/7
SAF (AU)	1.45 (±0.18)	1.63 (±0.31)	1.75 (±0.33)	1.85 (±0.32)	2.14 (±0.35)	2.28 (±0.56)	1.89 (±0.41)
Men	1.40 (±0.15)	1.61 (±0.30)	1.72 (±0.33)	1.84 (±0.32)	2.18 (±0.34)	2.57 (±0.68)	1.91 (±0.46)
Women	1.47 (±0.20)	1.64 (±0.32)	1.79 (±0.34)	1.85 (±0.32)	2.13 (±0.36)	2.03 (±0.23)	1.88 (±0.38)
Smoker	1.30 (±0.21)	1.88 (±0.46)	1.94 (±0.33)	1.86 (±0.24)	2.07 (±0.50)	3.05 (±1.49)	1.94 (±0.50)
Ex-smoker	1.65	1.46 (±0.32)	1.77 (±0.15)	1.88 (±0.33)	2.18 (±0.35)	2.33 (±0.55)	2.06 (±0.42)
Non-smoker	1.47 (±0.17)	1.59 (±0.26)	1.73 (±0.34)	1.83 (±0.33)	2.11 (±0.32)	2.12 (±0,32)	1.81 (±0.36)
FST I-II	1.44 (±0.19)	1.59 (±0.33)	1.70 (±0.34)	1.80 (±0.25)	2.06 (±0.28)	2.15 (±0.32)	1.82 (±0.35)
FST III-IV	1.48 (±0.25)	1.69 (±0.27)	1.81 (±0.31)	1.90 (±0.37)	2.20 (±0.39)	2.35 (±0.66)	1.95 (±0.43)
FST V-VI	1.55	1.98 (±0.39)	-	2.00	2.57 (±0.52)	-	2.17 (±0.53)
P sex	0.584	0.658	0.436	0.877	0.512	0.008 *	0.489
P smoking status	0.295	0.007 *	0.410	0.820	0.522	0.073	<0.001 *
P skin phototype	0.859	0.136	0.215	0.224	0.014 *	0.336	0.002 *
95% LL	1.34	1.56	1.66	1.79	2.07	2.07	1.85
95% UL	1.56	1.70	1.84	1.91	2.21	2.50	1.93

P: Indicates the statistical significance of the differences in SAF levels across ANOVA of sex, smoking status, and skin phototype in each age group; LL: lower; UL: upper limit; FST: Fitzpatrick skin type; FST I: light/pale white; FST II: white/fair; FST III: medium/white to olive; FST IV: olive/moderate brown; FST V: brown/dark brown; FST VI: black/very dark brown to black; SAF: skin autofluorescence; (*) significant differences between the different categories of the independent variable (*p* < 0.05).

**Table 2:** Baseline characteristics of the studies included in the systematic review.

Characteristics of the Studies			Characteristics of Healthy Participants					
Reference	Population	Study Design	*N*	Mean Age	Age Ranges	Smoking	% Women	FST
EVasCu study, 2024	Spanish	Cross-sectional	390	42.02 ± 13.14	18–74	12.40% *	63.07%	I–V
Barreto J, et al., 2023 [48]	Brazilian	Cross-sectional and cases ^1^ and controls	736	44 ± 11.39	18–80	0%	55.2%	I–V
Paolillo FR, et al., 2019 [53]	Brazilian	Cross-sectional and cases ^2^ and controls	42	33.05 ± 9 **	20–50	0%	38.09%	I–IV
Isami F, et al., 2018 [49]	Japanese	Cross-sectional	10,946	NA	20–79	NA	77.22%	NE
Randag AC, et al., 2015 [38]	Dutch	Cross-sectional	32	49 ± 12	30–70	0%	100%	II–IV
Stürmer M, et al., 2015 [50]	German	Cross-sectional and cases ^3^ and controls	107	54.06 ± 15.5	13–96	15.89%	53.27%	NE
Simon Klenovics K, et al., 2014 [51]	Slovakian	Cross-sectional	1385	NA	0–77	10.25%	62%	NE
Koetsier M, et al., 2010 [29]	Dutch	Cross-sectional	428	38 ± 21	1–91	22%	57.94%	I–IV
Yue X, et al., 2010 [33]	Chinese	Cross-sectional	991	45.65 ± 15.62 **	10–89	17.96%	46.11%	NE ^5^
Lutgers H, et al., 2006 [52]	Dutch	Cross-sectional and cases ^4^ and controls	231	52 ± 17	40–80	30%	62%	NE ^5^

(^1^) Cases diagnosed with chronic kidney disease; (^2^) Cases diagnosed with cardiovascular diseases, such as insulin resistance, diabetes, stroke, stroke plus diabetes, hypertension, and use of medication; (^3^) Cases diagnosed with hypertension; (^4^) Cases diagnosed with type 2 diabetes; (^5^) Skin reflectance (R%) evaluated, which is significantly associated with the skin phototype; (*) The percentage of smokers was calculated for 387 participants, since 3 participants did not report their smoking status; (**) Mean age and standard deviation were calculated from combined reported data using the formula for combined mean and standard deviation from subsamples; FST: Fitzpatrick skin type; NA: not available; NE: not evaluated.

## Data Availability

The raw data supporting the conclusions of this article will be made available by the authors, without undue reservation.

## Supplementary Materials

The following supporting information can be downloaded at https://www.mdpi.com/article/10.3390/jcm14020474/s1: Figure S1: Formulas used for data combination; Figure S2: Flow chart of the study participants in the current study from the original EVasCu study; Figure S3: Association between skin autofluorescence and age; Figure S4: Association between skin autofluorescence and age groups; Figure S5: Mean skin autofluorescence per age group in men and women; Figure S6: Mean skin autofluorescence per age group in smokers, ex-smokers and non-smokers; Figure S7: Mean skin autofluorescence per age group in different skin phototypes; Figure S8: Synthesised results of the skin autofluorescence meta-analysis by age groups; Figure S9: Subgroup analysis according to the type of smoking status; Table S1: STROBE statement. Checklist of items that should be included in reports of cross-sectional studies; Table S2: PRISMA 2020 checklist; Table S3: MOOSE checklist; Table S4: The search strategy following the PICO strategy; Table S5: Statistical equations predicting skin autofluorescence based on age for different populations; Table S6: Quality assessment with the tool for observational cohort and cross-sectional studies of the National Heart, Lung and Blood Institute; Table S7: Subgroup analysis according to smoking status for age groups.
