# Supplementary material for: Reference Values of Skin Autofluorescence by Age Groups in Healthy Spanish Adults: Results from the EVasCu Study, a Systematic Review, and a Meta-Analysis
Source: J Clin Med. 2025 Jan 13;14(2):474. doi: 10.3390/jcm14020474 (PMC11766177; doi:10.3390/jcm14020474)
Supplement: Supplementary file 1 [file jcm-14-00474-s001.zip › jcm-3392273-supplementary.pdf]

Supplementary material

Abbreviations

AGEs: Advanced glycation end products.

AU: arbitrary units

CVD: Cardiovascular diseases.

EVA: Early vascular ageing.

FST: Fitzpatrick skin type.

FST I: Light/pale white skin.

FST II: White/Fair skin.

FST III: Medium/white to olive skin.

FST IV: Olive/Moderate Brown skin.

FST V: Brown/Dark Brown skin.

FST VI: Black/Very Dark Brown to Black skin.

MOOSE: Meta-analysis of Observational Studies in Epidemiology.

SAF: Skin autofluorescence.

STROBE: Strengthening the Reporting of Observational Studies in Epidemiology.

Table S1. STROBE Statement. Checklist of items that should be included in reports of cross-sectional studies.

CHECKLIST STROBE	ITEM	RECOMMENDATION	PAGE
**Title and abstract**	1	(*a*) Indicate the study's design with a commonly used term in the title or the abstract	1
		(*b*) Provide in the abstract an informative and balanced summary of what was done and what was found	1
		**INTRODUCTION**	
**Background/rationale**	2	Explain the scientific background and rationale for the investigation being reported	2,3
**Objectives**	3	State specific objectives, including any prespecified hypotheses	3
		**METHODS**	
**Study design**	4	Present key elements of study design early in the paper	3
**Setting**	5	Describe the setting, locations, and relevant dates, including periods of recruitment, exposure, follow-up, and data collection	3
**Participants**	6	(*a*) Give the eligibility criteria, and the sources and methods of selection of participants	3
**Variables**	7	Clearly, define all outcomes, exposures, predictors, potential confounders, and effect modifiers. Give diagnostic criteria, if applicable	4
**Data sources/measurement**	8	For each variable of interest, give sources of data and details of methods of assessment (measurement). Describe comparability of assessment methods if there is more than one group	4
**Bias**	9	Describe any efforts to address potential sources of bias	4
**Study size**	10	Explain how the study size was arrived at	3
**Quantitative variables**	11	Explain how quantitative variables were handled in the analyses. If applicable,	4

		describe which groupings were chosen and why	
**Statistical methods**	12	(*a*) Describe all statistical methods, including those used to control for confounding	4
		(*b*) Describe any methods used to examine subgroups and interactions	4
		(*c*) Explain how missing data were addressed	4
		(*d*) If applicable, describe analytical methods taking account of sampling strategy	NA
		(*e*) Describe any sensitivity analyses	NA
		**RESULTS**	
**Participants**	13	(a) Report numbers of individuals at each stage of study—e.g. numbers potentially eligible, examined for eligibility, confirmed eligible, included in the study, completing follow-up, and analysed	6, Figure S2
		(b) Give reasons for non-participation at each stage	6, Figure S2
		(c) Consider use of a flow diagram	6, Figure S2
**Descriptive data**	14	(a) Give characteristics of study participants (e.g. demographic, clinical, social) and information on exposures and potential confounders	6, Table 1
		(b) Indicate number of participants with missing data for each variable of interest	6, Figure S2
**Outcome data**	15	Report numbers of outcome events or summary measures	6, Table 1
**Main results**	16	(*a*) Give unadjusted estimates and, if applicable, confounder-adjusted estimates and their precision (e.g., 95% confidence interval). Make clear which confounders were adjusted for and why they were included	6, Table 1
		(*b*) Report category boundaries when continuous variables were categorised	6, Table 1

		(*c*) If relevant, consider translating estimates of relative risk into absolute risk for a meaningful time period	NA
**Other analyses**	17	Report other analyses done—e.g. analyses of subgroups and interactions, and sensitivity analyses	7, Table 1, Figure S4, Figure S5, Figure S6, Figure S7
		**DISCUSSION**	
**Key results**	18	Summarise key results with reference to study objectives	11
**Limitations**	19	Discuss limitations of the study, taking into account sources of potential bias or imprecision. Discuss both direction and magnitude of any potential bias	13
**Interpretation**	20	Give a cautious overall interpretation of results considering objectives, limitations, multiplicity of analyses, results from similar studies, and other relevant evidence	11-13
**Generalisability**	21	Discuss the generalisability (external validity) of the study results	12,13
		**OTHER INFORMATION**	
**Funding**	22	Give the source of funding and the role of the funders for the present study and, if applicable, for the original study on which the present article is based	13

Table S2. PRISMA 2020 Checklist.

Section and Topic	Item #	Checklist item	Location where item is reported
**TITLE**			
Title	1	Identify the report as a systematic review.	1
**ABSTRACT**			
Abstract	2	See the PRISMA 2020 for Abstracts checklist.	1
**INTRODUCTION**			
Rationale	3	Describe the rationale for the review in the context of existing knowledge.	2,3
Objectives	4	Provide an explicit statement of the objective(s) or question(s) the review addresses.	3
**METHODS**			
Eligibility criteria	5	Specify the inclusion and exclusion criteria for the review and how studies were grouped for the syntheses.	5
Information sources	6	Specify all databases, registers, websites, organisations, reference lists and other sources searched or consulted to identify studies. Specify the date when each source was last searched or consulted.	5
Search strategy	7	Present the full search strategies for all databases, registers and websites, including any filters and limits used.	5, Table S4
Selection process	8	Specify the methods used to decide whether a study met the inclusion criteria of the review, including how many reviewers screened each record and each report retrieved, whether they worked independently, and if applicable, details of automation tools used in the process.	5
Data collection process	9	Specify the methods used to collect data from reports, including how many reviewers collected data from each report, whether they worked independently, any processes for obtaining or confirming data from study investigators, and if applicable, details of automation tools used in the process.	5
Data items	10a	List and define all outcomes for which data were sought. Specify whether all results that were compatible with each outcome domain in each study were sought (e.g. for all measures, time points, analyses), and if not, the methods used to decide which results to collect.	5
	10b	List and define all other variables for which data were sought (e.g. participant and intervention characteristics, funding sources). Describe any assumptions made about any missing or unclear information.	5
Study risk of bias assessment	11	Specify the methods used to assess risk of bias in the included studies, including details of the tool(s) used, how many reviewers assessed each study and whether they worked independently, and if applicable, details of automation tools used in the process.	5
Effect measures	12	Specify for each outcome the effect measure(s) (e.g. risk ratio, mean difference) used in the synthesis or presentation of results.	5
Synthesis methods	13a	Describe the processes used to decide which studies were eligible for each synthesis (e.g. tabulating the study intervention characteristics and comparing against the planned groups for each synthesis (item #5)).	5
	13b	Describe any methods required to prepare the data for presentation or synthesis, such as handling of missing summary statistics, or data conversions.	5
	13c	Describe any methods used to tabulate or visually display results of individual studies and syntheses.	5,6
	13d	Describe any methods used to synthesize results and provide a rationale for the choice(s). If meta-analysis was performed, describe the model(s), method(s) to identify the presence and extent of statistical heterogeneity, and software package(s) used.	5,6

Section and Topic	Item #	Checklist item	Location where item is reported
	13e	Describe any methods used to explore possible causes of heterogeneity among study results (e.g. subgroup analysis, meta-regression).	6
	13f	Describe any sensitivity analyses conducted to assess robustness of the synthesized results.	6
Reporting bias assessment	14	Describe any methods used to assess risk of bias due to missing results in a synthesis (arising from reporting biases).	NA
Certainty assessment	15	Describe any methods used to assess certainty (or confidence) in the body of evidence for an outcome.	NA
**RESULTS**			
Study selection	16a	Describe the results of the search and selection process, from the number of records identified in the search to the number of studies included in the review, ideally using a flow diagram.	6, 7, Figure 1
	16b	Cite studies that might appear to meet the inclusion criteria, but which were excluded, and explain why they were excluded.	Figure 1
Study characteristics	17	Cite each included study and present its characteristics.	Table 2
Risk of bias in studies	18	Present assessments of risk of bias for each included study.	9, Table S6
Results of individual studies	19	For all outcomes, present, for each study: (a) summary statistics for each group (where appropriate) and (b) an effect estimate and its precision (e.g. confidence/credible interval), ideally using structured tables or plots.	Table 1, Figure 2, Figure S9
Results of syntheses	20a	For each synthesis, briefly summarise the characteristics and risk of bias among contributing studies.	9
	20b	Present results of all statistical syntheses conducted. If meta-analysis was done, present for each the summary estimate and its precision (e.g. confidence/credible interval) and measures of statistical heterogeneity. If comparing groups, describe the direction of the effect.	9, 10, Figure 2, Figure S9
	20c	Present results of all investigations of possible causes of heterogeneity among study results.	11
	20d	Present results of all sensitivity analyses conducted to assess the robustness of the synthesized results.	11
Reporting biases	21	Present assessments of risk of bias due to missing results (arising from reporting biases) for each synthesis assessed.	NA
Certainty of evidence	22	Present assessments of certainty (or confidence) in the body of evidence for each outcome assessed.	NA
**DISCUSSION**			
Discussion	23a	Provide a general interpretation of the results in the context of other evidence.	11, 12
	23b	Discuss any limitations of the evidence included in the review.	13
	23c	Discuss any limitations of the review processes used.	13
	23d	Discuss implications of the results for practice, policy, and future research.	12, 13
**OTHER INFORMATION**			
Registration and protocol	24a	Provide registration information for the review, including register name and registration number, or state that the review was not registered.	5
	24b	Indicate where the review protocol can be accessed, or state that a protocol was not prepared.	5
	24c	Describe and explain any amendments to information provided at registration or in the protocol.	NA
Support	25	Describe sources of financial or non-financial support for the review, and	14

Section and Topic	Item #	Checklist item	Location where item is reported
		the role of the funders or sponsors in the review.	
Competing interests	26	Declare any competing interests of review authors.	14
Availability of data, code and other materials	27	Report which of the following are publicly available and where they can be found: template data collection forms; data extracted from included studies; data used for all analyses; analytic code; any other materials used in the review.	NA

From: Page MJ, McKenzie JE, Bossuyt PM, Boutron I, Hoffmann TC, Mulrow CD, et al. The PRISMA 2020 statement: an updated guideline for reporting systematic reviews. BMJ 2021;372:n71. doi: 10.1136/bmj.n71

Table S3. MOOSE Checklist.

Item No	Recommendation	Reported on Page No
Reporting of background should include		
1	Problem definition	1
2	Hypothesis statement	1
3	Description of study outcome(s)	1
4	Type of exposure or intervention used	1
5	Type of study designs used	1
6	Study population	1
Reporting of search strategy should include		
7	Qualifications of searchers (eg, librarians and investigators)	5
8	Search strategy, including time period included in the synthesis and key words	5
9	Effort to include all available studies, including contact with authors	5
10	Databases and registries searched	5
11	Search software used, name and version, including special features used (eg, explosion)	5
12	Use of hand searching (eg, reference lists of obtained articles)	5
13	List of citations located and those excluded, including justification	NA
14	Method of addressing articles published in languages other than English	NA
15	Method of handling abstracts and unpublished studies	5
16	Description of any contact with authors	NA
Reporting of methods should include		
17	Description of relevance or appropriateness of studies assembled for assessing the hypothesis to be tested	5
18	Rationale for the selection and coding of data (eg, sound clinical principles or convenience)	5
19	Documentation of how data were classified and coded (eg, multiple raters, blinding and interrater reliability)	5
20	Assessment of confounding (eg, comparability of cases and controls in studies where appropriate)	5
21	Assessment of study quality, including blinding of quality assessors, stratification or regression on possible predictors of study results	5
22	Assessment of heterogeneity	6
23	Description of statistical methods (eg, complete description of fixed or random effects models, justification of whether the chosen models account for predictors of study results, dose-response models, or cumulative meta-analysis) in sufficient detail to be replicated	5, 6
24	Provision of appropriate tables and graphics	5, 6
Reporting of results should include		
25	Graphic summarizing individual study estimates and overall estimate	Figure 2
26	Table giving descriptive information for each study included	Table 2
27	Results of sensitivity testing (eg, subgroup analysis)	10, Figure S9
28	Indication of statistical uncertainty of findings	10, 11

Item No	Recommendation	Reported on Page No
Reporting of discussion should include		
29	Quantitative assessment of bias (eg, publication bias)	10
30	Justification for exclusion (eg, exclusion of non-English language citations)	Figure 1
31	Assessment of quality of included studies	9
Reporting of conclusions should include		
32	Consideration of alternative explanations for observed results	13, 14
33	Generalization of the conclusions (ie, appropriate for the data presented and within the domain of the literature review)	13, 14
34	Guidelines for future research	14
35	Disclosure of funding source	14

From: Stroup DF, Berlin JA, Morton SC, et al, for the Meta-analysis Of Observational Studies in Epidemiology (MOOSE) Group. Meta-analysis of Observational Studies in Epidemiology. A Proposal for Reporting. *JAMA*. 2000;283(15):2008-2012. doi: 10.1001/jama.283.15.2008.

Table S4. The search strategy following the PICO strategy.

Health* **OR** "Apparent healthy" **OR** "Without disease" **OR** "Control group" **OR** "Control subject*" **OR** "population" **OR** "Healthy population" **OR** "Healthy adults" **OR** "Healthy people"	**AND**	"Skin autofluorescence" **OR** "Skin auto-fluorescence" **OR** SAF **OR** AF	**AND**	"Glycation end products" **OR** "Glycation end-products" **OR** "Advanced glycation end products" **OR** "Advanced glycation end-products"	**AND**	"Reference value*" **OR** "Reference range*" **OR** "Mean SAF" OR "Mean AF" OR "Mean skin auto*" OR "Normal value*" OR "Normal range*" OR "SAF values" OR "Skin autofluorescence values" OR "AF values"

Table S5. Statistical equations for predicting skin autofluorescence in different populations on the basis of age.

Reference	Population	Equation for SAF
EVasCu, et al, 2024	Spanish	$SAF = 1.149+0.018*age$
		$SAF_{women} = 1.263+0.015*age$
		$SAF_{men} = 0.914+0.002*age$
		$SAF_{smoker} = 1.379+0.014*age$
		$SAF_{ex\text{-}smoker} = 0.903+0.023*age$
		$SAF_{non\text{-}smoker} = 1.187+0.016*age$
		$SAF_{FST\text{-}I\text{-}II} = 1.180+0.015*age$
		$SAF_{FST\text{-}III\text{-}IV} = 1.176+0.018*age$
		$SAF_{FST\text{-}V} = 1.279+0.022*age$
Barreto J, et al, 2023 [48]	Brazilian	$SAFwomen = 1.22+0.020*age$
		$SAFmen = 0.87+0.026*age$
Isami F, et al, 2018 [49]	Japanese	$SAF = (132.23+1.38*age)/100$
Randag AC, et al, 2015 [38]	Dutch	$SAF = 0.731+0.0183*age$
Simon-Klenovics K, et al, 2014 [51]	Slovakian	$SAF^1 = 0.76+0.022*age$
Koetsier M, et al, 2010 [29]	Dutch	$SAF^1 = 0.83+0.023*age$
Yue X, et al, 2010 [33]	Chinese	$SAF = 1.230+0.012*age$

SAF: Skin autofluorescence. ([1]) The studies included a population under 18 years of age in the analyses to determine the predictive formula for SAF.

Table S6. Quality assessment with the tool for observational cohort and cross-sectional studies of the National Heart, Lung and Blood Institute.

Reference	1	2	3	4	5	6	7	8	9	10	11	12	13	14	Overall
EVasCu study, 2024	Y	Y	Y	Y	Y	Y	NA	Y	Y	NA	Y	NR	NA	Y	GOOD
Barreto, J, et al, 2023[48]	Y	Y	Y	Y	NR	Y	NA	Y	Y	NA	Y	NR	NA	Y	GOOD
Paolillo, FR,et al, 2019[53]	Y	Y	Y	Y	NR	Y	NA	Y	Y	NA	Y	NR	NA	Y	GOOD
Isami F, et al, 2018[49]	Y	Y	Y	Y	NR	Y	NA	Y	Y	NA	Y	NR	NA	Y	GOOD
Randag, AC, et al, 2015[38]	Y	Y	Y	Y	NR	Y	NA	Y	Y	NA	Y	NR	NA	Y	GOOD
Stürmer, M, et al., 2015[50]	Y	Y	Y	Y	NR	Y	NA	Y	Y	NA	Y	NR	NA	Y	GOOD
Simon Klenovics K, et al, 2014[51]	Y	Y	Y	Y	NR	Y	NA	Y	Y	NA	Y	NR	NA	Y	GOOD
Koetsier M, et al, 2010[29]	Y	Y	Y	Y	NR	Y	NA	Y	Y	NA	Y	NR	NA	Y	GOOD
Yue X, et al, 2010[33]	Y	Y	Y	Y	NR	Y	NA	Y	Y	NA	Y	NR	NA	Y	GOOD
Lutgers H, et al, 2006[52]	Y	Y	Y	Y	Y	Y	NA	Y	Y	NA	Y	NR	NA	Y	GOOD

Assessment of risk of bias for observational studies with 'Study Quality Assessment Tools'. Green: yes (Y); Red: no (N); Yellow: cannot determine (CD), not reported (NR); White: not applicable (NA),

<u>Items for Study Quality Assessment Tools:</u>

1. Was the research question or objective in this paper clearly stated?
2. Was the study population clearly specified and defined?
3. Was the participation rate of eligible persons at least 50%?
4. Were all the subjects selected or recruited from the same or similar populations (including the same time period)? Were inclusion and exclusion criteria for being in the study prespecified and applied uniformly to all participants?
5. Was a sample size justification, power description, or variance and effect estimates provided?
6. For the analyses in this paper, were the exposure(s) of interest measured prior to the outcome(s) being measured?
7. Was the timeframe sufficient so that one could reasonably expect to see an association between exposure and outcome if it existed?
8. For exposures that can vary in amount or level, did the study examine different levels of the exposure as related to the outcome (e.g., categories of exposure, or exposure measured as continuous variable)?
9. Were the exposure measures (independent variables) clearly defined, valid, reliable, and implemented consistently across all study participants?
10. Was the exposure(s) assessed more than once over time?
11. Were the outcome measures (dependent variables) clearly defined, valid, reliable, and implemented consistently across all study participants?
12. Were the outcome assessors blinded to the exposure status of participants?
13. Was loss to follow-up after baseline 20% or less?
14. Were key potential confounding variables measured and adjusted statistically for their impact on the relationship between exposure(s) and outcome(s)?
15. Overall bias: good, fair, or poor. If poor, it need a justification.

Table S7. Subgroup analysis according to smoking status across age groups.

Covariable	N Studies	Mean (95% CI)	I^2 (%)	p-value
**Smokers**				
Group 20-29	3	1.64 (1.37,1.92)	97.7%	0.000
Group 30-39	3	1.86 (1.50, 2.23)	79.0%	0.009
Group 40-49	3	1.82 (1.75, 1.90)	0.0%	0.588
Group 50-59	3	2.17 (1.93, 2.42)	55.2%	0.107
Group ≥60	3	2.59 (2.45, 2.73)	0.0%	0.896
**Non-smokers**				
Group 20-29	4	1.52 (1.31, 1.74)	95.8%	0.000
Group 30-39	6	1.67 (1.48, 1.86)	96.3%	0.000
Group 40-49	6	1.88 (1.71, 2.05)	95.7%	0.000
Group 50-59	5	2.11 (1.96, 2.26)	87.6%	0.000
Group ≥60	5	2.26 (2.00, 2.52)	94.4%	0.000

I^2 = Statistic I; N= Number of studies; p-value: Indicates the statistical significance of I^2 for assessing heterogeneity on the basis of the means and confidence intervals used in the meta-analysis.

Table S8. Main factors with the potential to exert influence upon skin autofluorescence values.

**Factors related to the individual**	
Modifiables	Smoking status, body mass index[1], physical activity, duration and quality of sleep, variation and composition of diet, alcohol consumption, exposure to environmental contaminants[1], sunlight exposure[1], place of residence[1], type of work performed[1], socioeconomic status[1], etc.
Non-modifiables	Age, sex, skin fototype[2], ethnicity, genetic determinants, familiar incidence of CVD, hormonal status[2], poor glycemic control, adiposity or metabolic dysregulation[2], oxidative stress[2], chronic inflammation[2], chronic disease (cardiovascular, renal, degenerative…), dyslipidemia[2], etc.
**Factors related to measurement**	
Controllables	Room temperature (between 22–24°C), room humidity (33–65%), room light (dark or semi-dark environment), skin cleanliness (presence of creams, makeup, dirt, etc.), skin hydration, measurement area, presence of increased pigmentation (tattoos, moles, spots, scars, etc), etc.

([1]) These factors are challenging to modify and can, at times, be regarded as non-modifiable. ([2]) These factors may be amenable to modification, they are, as a general rule, not.

$$\bar{x}_c = \frac{\sum_{i=1}^{k}(n_i \cdot \bar{x}_i)}{\sum_{i=1}^{k} n_i} \qquad s_c = \sqrt{\frac{\sum_{i=1}^{k}(n_i \cdot 1)s_i^2}{\sum_{i=1}^{k}(n_i - k)}}$$

$\bar{x}_c$= Combined subgroups mean
s_c= Combined subgroups standard deviation
$\bar{x}$= Mean of subgroup
s= Standard deviation of subgroup
i= Subgroup index
k= Total number of subgroups

Figure S1. Formulas used for data combination.

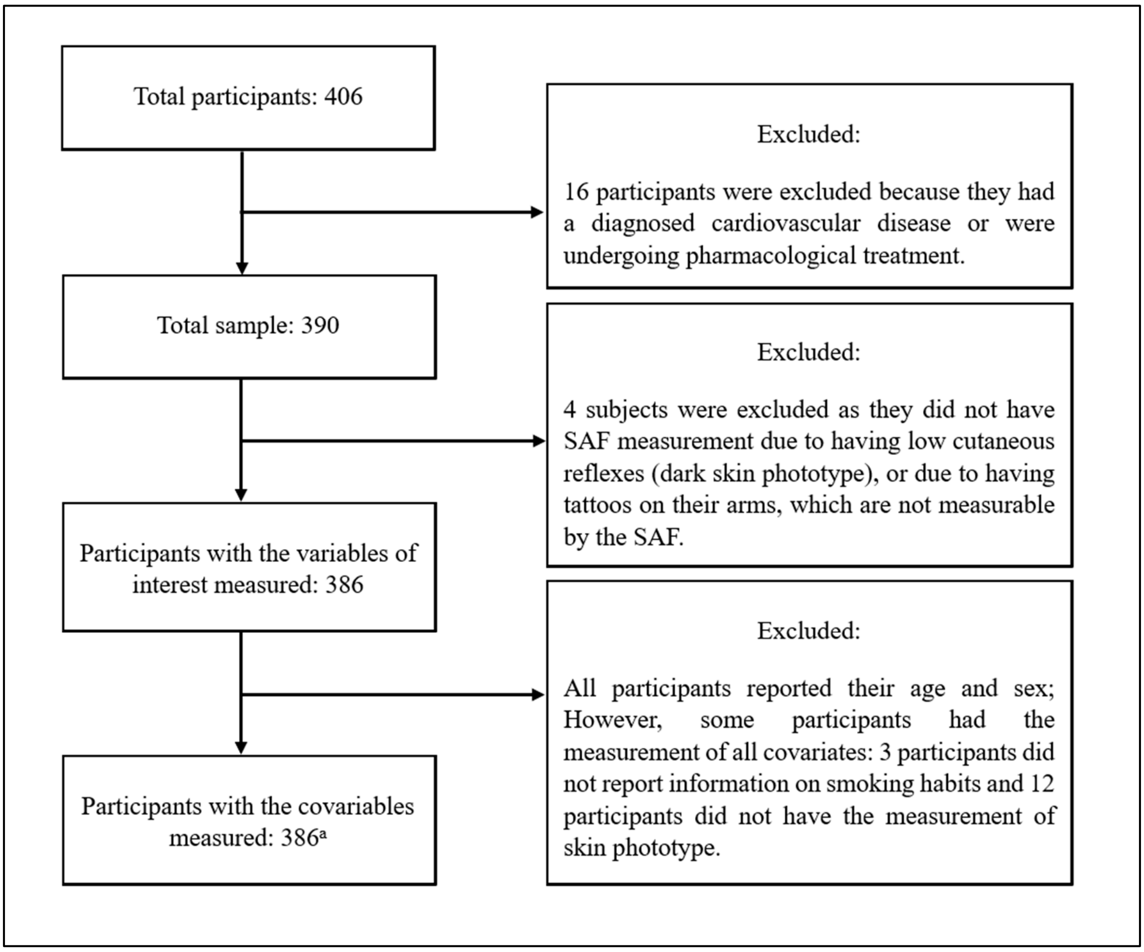

Figure S2. Flowchart of the study participants in the current study from the original EVasCu study.

(a) Not all participants had all the covariates measured; in the case of age and sex, all participants reported this information. However, some participants did not have all the covariates measured: 3 participants did not report information on smoking status, and 12 participants had no skin phototype recorded.

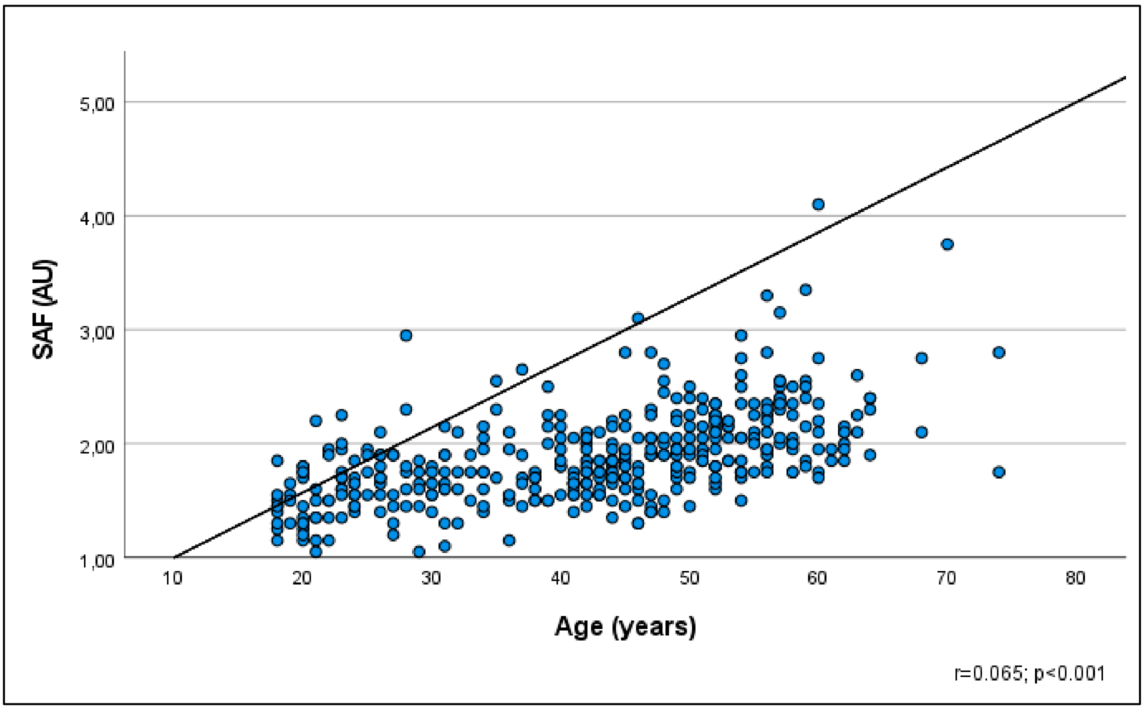

Figure S3. Association between skin autofluorescence and age.

AU: arbitrary units; p: statistical significance of the correlation between SAF score and age; r: Pearson correlation coefficient between SAF score and age; SAF: skin autofluorescence.

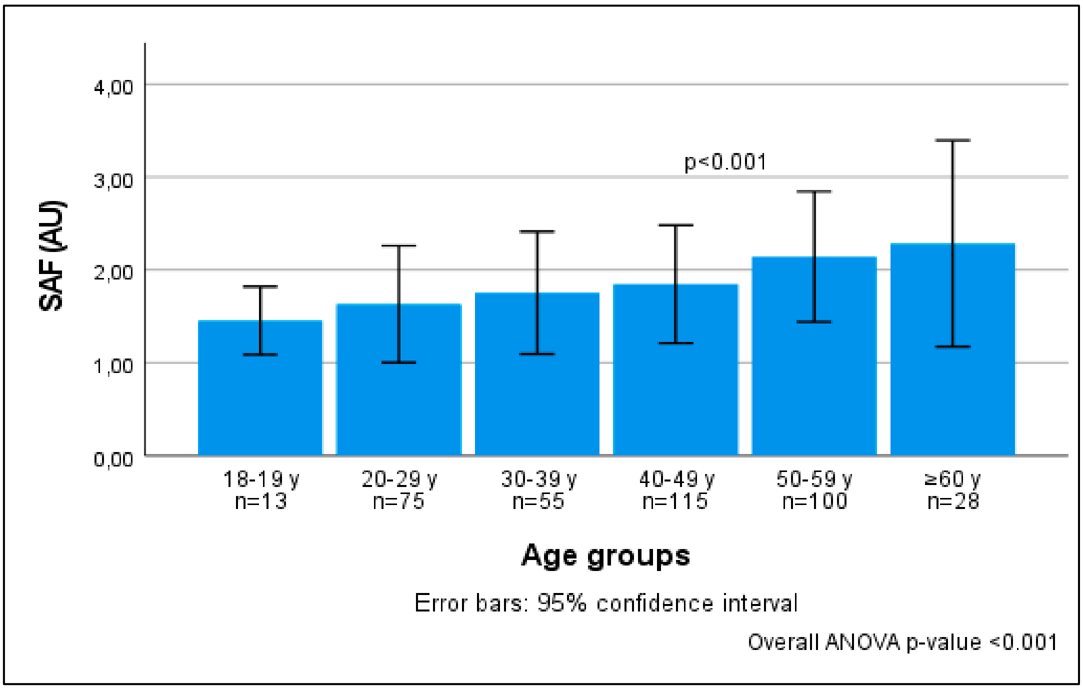

Figure S4. Association between skin autofluorescence and age groups.

AU: arbitrary units; Overall ANOVA p-value: Indicates the statistical significance of the differences in SAF levels across different age groups; p: Indicates significant differences between consecutive age groups, as determined by Student's t-test; SAF: skin autofluorescence; y: years.

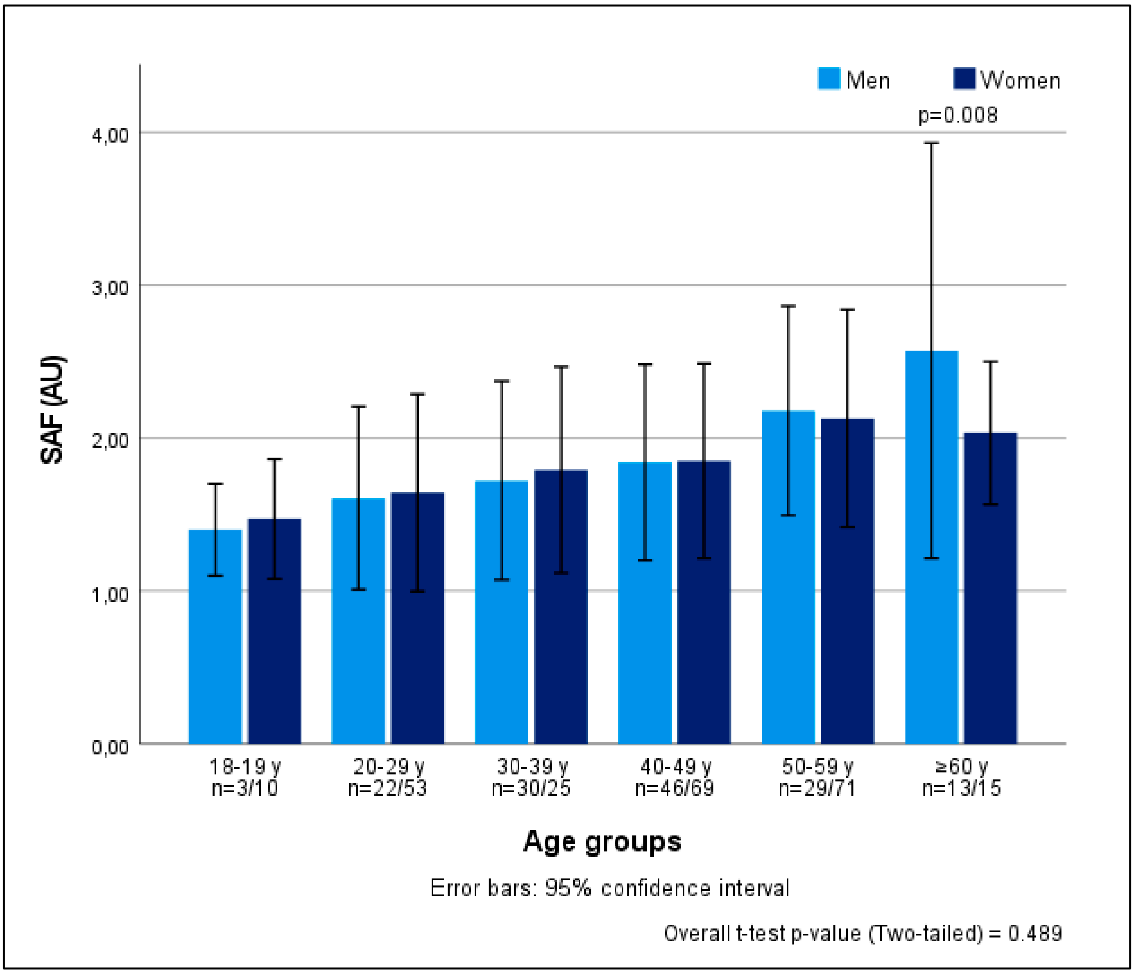

Figure S5. Mean skin autofluorescence per age group in men and women.

Among the 386 subjects who had the SAF measure, 37.05% were men and 62.95% were women, with an association between sex and age groups ($\chi 2$: 14.485; p = 0.013). Women tended to present a greater SAF than men did until age 49, with the trend reversing after age 50, where there were significant differences between sexes in 60 years and older age group (p=0.008). The equation for men would be y= 0.914 + 0.023x age (R2=39.4%; F=91.56; p=<0.001), and for women, it would be y=1.263+0.015 x age (R2= 27.8%; F=91.56, p=<0.001).

AU: Arbirary units; n: number of subjects (men/woman); Overall t-test p-value (two-tailed): Significance of the difference in the SAF score between men and women subjects, as determined by a two-tailed Student's t-test; p: Indicates significant differences in sex (men and women) within each age group, as determined by Student’s t-test. SAF: Skin autofluorescence; y: years.

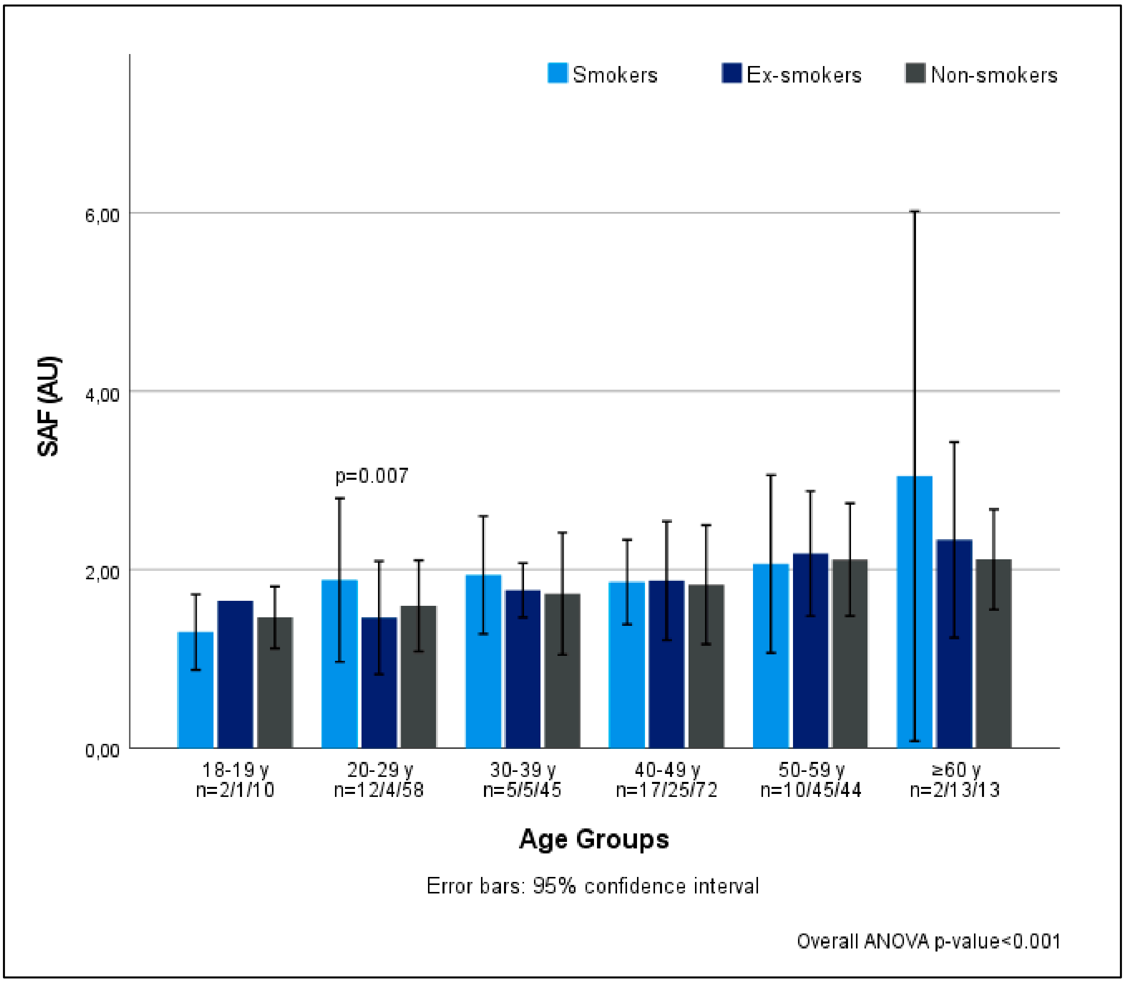

Figure S6. Mean skin autofluorescence per age group in smokers, ex-smokers and non-smokers.

Among the 383 subjects who reported their smoking status, 12.5% were smokers, 24.5% were ex-smokers and 63.2% were non-smokers, with an association between smoking status and age groups ($\chi2$: 57,649; $p < 0.001$). In terms of sex, 14.1% of women and 9.9% of men were smokers, 22.4% of women and 27.5% of men were ex-smokers, and 63.5% of men were smokers. A total of 62.7% of the women and 62.7% of the men were non-smokers, with no association between sex and smoking ($\chi2$: 2.238; $p = 0.327$). The non-smoking group followed a linear increase in the SAF score with increasing age; however, for ex-smokers and smokers, the SAF score did not follow the same trend across all groups. For ex-smokers, the increase in SAF increased with age, except in the 18–19 years age group, where there was only a single ex-smoker subject. For smokers, there was also an increase in the SAF score with increasing age, except in the 40–49 years group. Compared with ex-smokers, smokers presented higher mean SAF values, which, in turn, were greater than those of non-smokers in the 30–39 years age group and in those over 60 years and older. The other groups do not follow this trend, following different patterns, ex-smokers in the 18–19 years, 40–49 years and 50–59 years age groups obtained the highest SAF values, while the highest SAF values among smokers in the 20–29 years age group.

AU: arbitrary units; n: number of subjects (smokers/ex-smokers/non-smokers); Overall ANOVA p-value: Indicates the statistical significance of the differences in SAF levels across different smoking statuses; p: Indicates significant differences among the three groups of smoking statuses (smokers, ex-smokers and non-smokers) within each age group, as determined by ANOVA. SAF: Skin autofluorescence; y: years.

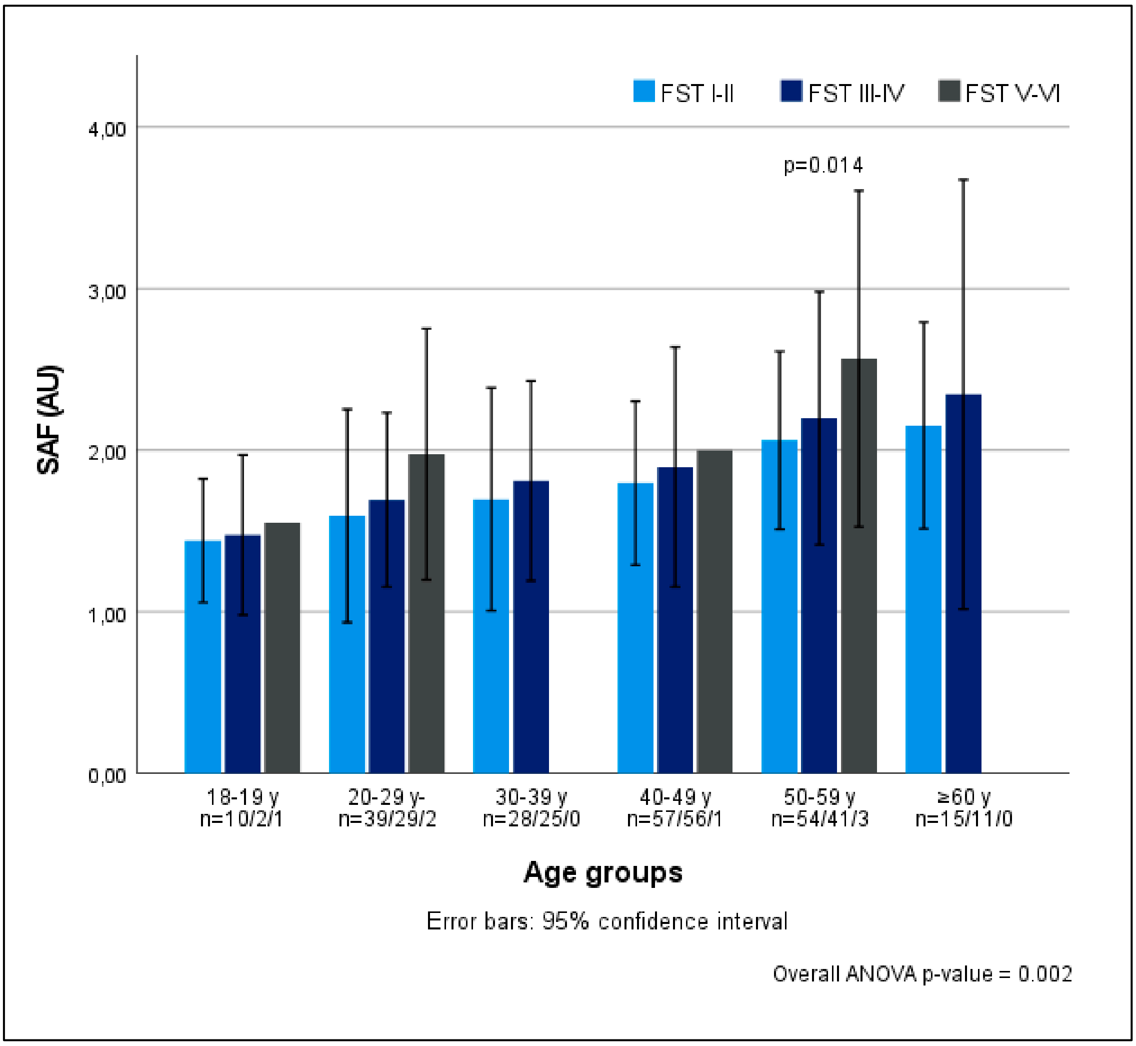

Figure S7. Mean skin autofluorescence per age group for different skin phototypes.

Among the 374 subjects whose skin phototype was measured through the Fitzpatrick questionnaire (FST), 54.3% had a FST of I-II, 43.9% had a FST of III-IV, and 1.9% had a FST of V, indicating that there was no association between the skin phototype and age group ($\chi2$: 10.723; p = 0.380). With respect to sex, 58.5% of women and 47.1% of men had a skin phototype of I-II, 40.3% of women and 50% of men had a FST of III-IV, and 1.3% of women and 2.9% of men had a FST of V, with no association found between sex and FST ($\chi2$: 5.193; p = 0.075). Despite the lack of an apparent association between FST and age group, a trend of increasing SAF was shown as the skin phototype increased in all age groups, with darker skin phototypes resulting in higher values.

AU: arbitrary units; FST I-II: Light/Pale white/White/Faire skin phototypes; FST III-IV: Medium/White to olive/Olive/Moderate brown skin phototypes; FST V-VI: Brown/Dark Brown/Black/Very Dark Brown to Black skin phototypes. n: number of subjects with respect to FST categories (FST I-II/FST III-IV/FST V/VI); Overall ANOVA p-value: Indicates the statistical significance of the differences in SAF levels across different FST; p: Indicates significant differences among the three groups of FST (FST I-II, FST III-IV, and FST V-VI) within each age group, as determined by ANOVA; SAF: Skin autofluorescence; y: years.

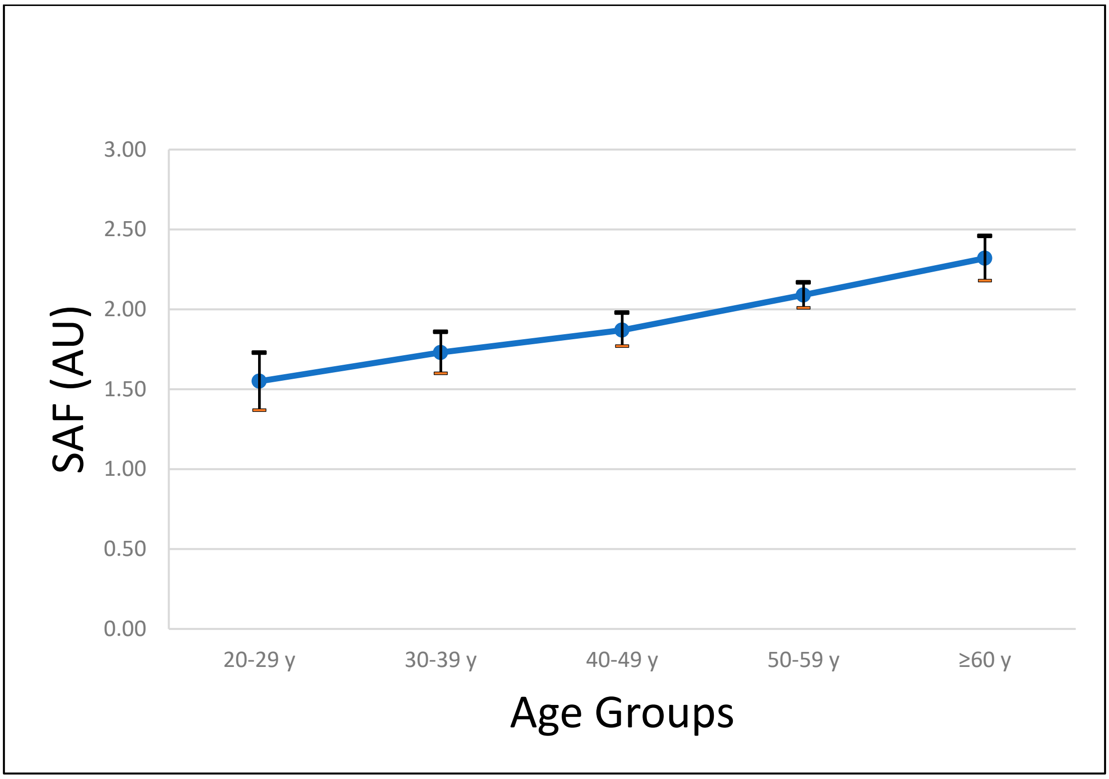

Figure S8. Synthesised results of the skin autofluorescence meta-analysis by age groups.

AU: arbitrary units; SAF: skin autofluorescence; y: years.

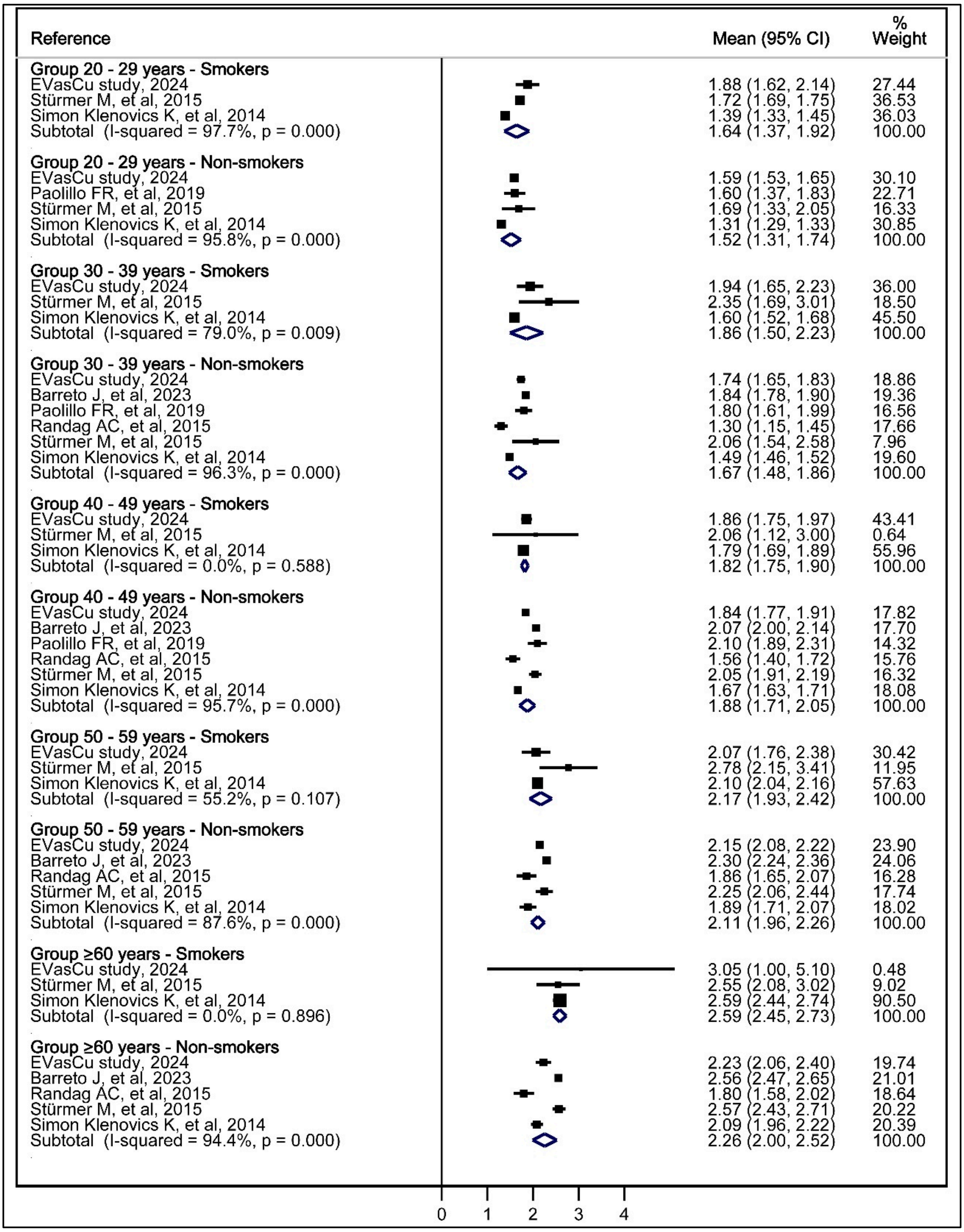

Figure S9. Subgroup analysis of mean skin autofluorescence according to smoking status.
